# Preliminary analysis of a multicenter study of Pola-R-CHP in untreated Japanese patients with DLBCL (POLASTAR)

**DOI:** 10.1007/s12185-025-04122-w

**Published:** 2025-12-03

**Authors:** Atsushi Satake, Yasuhiro Nagate, Kohta Miyawaki, Yuki Fujiwara, Shuichi Ota, Tsuyoshi Muta, Shinya Rai, Hisashi Tsurumi, Ritsuro Suzuki, Takaaki Miyake, Hideki Goto, Noriko Fukuhara, Mamiko Sakata-Yanagimoto, Koji Izutsu, Momoko Nishikori, Hirohiko Shibayama, Takahiro Kumode, Daisuke Ennishi, Takayuki Shimose, Naoki Inubashiri, Itaru Matsumura, Koichi Akashi, Koji Kato

**Affiliations:** 1 Kansai Medical University, Hirakata, Japan; 2 NHO Osaka National Hospital, Osaka, Japan; 3 Kyushu University Graduate School of Medical Sciences, Fukuoka, Japan; 4 Japanese Red Cross Society Himeji Hospital, Himeji, Japan; 5 Sapporo Hokuyu Hospital, Sapporo, Japan; 6 Hiroshima Red Cross Hospital, Hiroshima, Japan; 7 Kindai University, Osaka, Japan; 8 Matsunami General Hospital, Kasamatsu, Japan; 9 Shimane University Hospital, Izumo, Japan; 10 Shimane Prefectural Central Hospital, Izumo, Japan; 11 Hokkaido University Hospital, Sapporo, Japan; 12 Tohoku University, Sendai, Japan; 13 University of Tsukuba, Tsukuba, Japan; 14 National Cancer Center Hospital, Tokyo, Japan; 15 Kyoto University Hospital, Kyoto, Japan; 16 Okayama University Hospital, Okayama, Japan; 17 Clinical Research Support Center Kyushu, Fukuoka, Japan; 18 Chugai Pharmaceutical Co., Ltd., Tokyo, Japan

**Keywords:** DLBCL, Elderly patients, Pola-R-CHP, Polatuzumab vedotin, Real-world data

## Abstract

Polatuzumab vedotin plus rituximab, cyclophosphamide, doxorubicin, and prednisone (Pola-R-CHP) was approved in Japan for the treatment of previously untreated diffuse large B-cell lymphoma (DLBCL) in 2022, based on findings of the POLARIX study (NCT03274492). Reports on real-world usage of Pola-R-CHP are lacking. Here we report safety and response rates at end of treatment (EOT) for Pola-R-CHP in Japan from the real-world observational POLASTAR study (jRCT1071220082). Patients (≥ 18 years) with previously untreated DLBCL who were scheduled to receive Pola-R-CHP were enrolled. The primary endpoint was overall survival. As of December 20, 2023, the full analysis set (FAS) included 192 of the initial 199 patients enrolled. Median age was 71.0 years (range 30–91). In the FAS, 99 (51.6%) patients had Grade ≥ 3 adverse events (AEs), 29 (15.1%) had serious AEs, and 15 (7.8%) discontinued polatuzumab vedotin due to AEs. In the efficacy-evaluable population at EOT (*n* = 141), the overall response rate was 95.0% [95% confidence interval (CI), 90.1–97.6], and the complete response rate was 87.9% (95% CI, 81.5–92.3). These results are consistent with published data from POLARIX. The POLASTAR study is ongoing; the recruitment target of 500 patients has been reached.

**Clinical trial registration:** Japan Registry of Clinical Trials (jRCT1071220082).

**Supplementary Information:**

The online version contains supplementary material available at 10.1007/s12185-025-04122-w.

## Introduction

Diffuse large B-cell lymphoma (DLBCL) is the most common type of non-Hodgkin lymphoma (NHL), accounting for 30–58% of NHL cases in developed countries and an even higher percentage in developing countries [1, 2]. While approximately 60% of patients with previously untreated DLBCL can be cured by rituximab, cyclophosphamide, doxorubicin, vincristine, and prednisone (R-CHOP), ~ 40% of patients do not respond to or relapse following initial R-CHOP therapy [3]. For the past 20 years, treatments being evaluated in various clinical trials, most following an “R-CHOP ± X” design, have failed to improve clinical outcomes compared with standard R-CHOP [4–6].

Polatuzumab vedotin is an antibody–drug conjugate composed of a humanized anti-CD79b monoclonal antibody, conjugated with a protease-cleavable linker to monomethyl auristatin E, a potent microtubule inhibitor [7, 8]. Polatuzumab vedotin in combination with bendamustine and rituximab has shown promising clinical outcomes in patients with relapsed/refractory DLBCL, leading to the approval of this regimen in the EU and US [9, 10], as well as in Japan as of March 2021 [11]. More recently, in the phase III, international, multicenter, randomized, double-blind, placebo-controlled POLARIX study (NCT03274492), polatuzumab vedotin in combination with rituximab plus cyclophosphamide, doxorubicin and prednisone (Pola-R-CHP) demonstrated a clinically meaningful improvement in progression-free survival versus R-CHOP in patients with previously untreated DLBCL, with a comparable safety profile [12]. Based on the findings from the POLARIX study, Pola-R-CHP was approved for the treatment of patients with previously untreated DLBCL in > 90 countries. Pola-R-CHP treatment is used in clinical practice in Japan, and current Japanese Practical guidelines for Hematological Malignancies recommend 6 cycles of Pola-R-CHP followed by 2 cycles of rituximab monotherapy for patients with DLBCL aged 18–80 years with an International Prognostic Index (IPI) score of ≥ 2 [13, 14]. Furthermore, Pola-R-CHP is reimbursed by public health insurance regardless of whether a patient meets the eligibility criteria of the POLARIX study.

The recently published POLARIX Asia subpopulation analysis demonstrated consistent efficacy and safety with Pola-R-CHP versus R-CHOP in the Asian subpopulation and the global POLARIX population [15]. The analysis included 281 Asian patients from POLARIX (China, *n* = 150; Japan, *n* = 85; Republic of Korea, *n* = 31; Taiwan, *n* = 15); however, among the 85 Japanese patients that were enrolled, only 44 received Pola-R-CHP. As such, available data to date do not sufficiently reflect real-world Pola-R-CHP usage in Japan, and more real-world studies with larger Japanese patient populations are required to overcome the limited information currently available. Obtaining additional real-world efficacy and safety data will also allow for the evaluation of patients who were ineligible for the POLARIX study, including elderly patients aged > 80 years old, the exploration of better biomarker candidates (e.g., circulating tumor [ct]DNA), and the improvement in management of adverse events (AEs).

This real-world observational POLASTAR study (jRCT1071220082) was designed to investigate the efficacy and safety of Pola-R-CHP in a clinical setting for patients with previously untreated DLBCL, as well as to explore predictors of early relapse and response to Pola-R-CHP that could potentially be used to inform personalized health care. In this preliminary analysis, we present demographic and baseline characteristics, safety and response at end of treatment (EOT) in patients enrolled in the POLASTAR study.

### Methods

#### Study design and patients

POLASTAR (jRCT1071220082) is a nationwide real-world, multicenter, prospective, observational study designed to assess the efficacy and safety of Pola-R-CHP in patients with previously untreated DLBCL. This analysis focuses primarily on reporting early safety profiles and response at end of treatment (EOT) in the initial 200 patients; data cut-off for this preliminary analysis was set at 24 weeks after enrollment of the 200th patient.

Patients were included if they were aged ≥ 18 years, had previously untreated DLBCL, and were scheduled to receive Pola-R-CHP per the latest package insert as part of their routine care. There were no restrictions on age, Eastern Cooperative Oncology Group performance status (ECOG PS) or IPI; patients were selected based on the physician’s choice. The planned observation period was 3 years after the first Pola-R-CHP administration in the last enrolled patient. All patients provided written informed consent.

Data were obtained from healthcare information and clinical diagnosis was carried out at each site; central review for diagnosis was not performed. Biological samples were collected during routine care; immunohistochemistry was performed at each site, and exploratory genetic analysis was conducted on biological samples but is not included in the current report. There were no restrictions on the use or extent of tests, medications, or other medical acts performed for diagnostic or therapeutic purposes. Treatment was provided according to the latest package insert and patients who received any component of Pola-R-CHP at a reduced dose from the first cycle, based on the physician’s decision, were also enrolled. There were no guidelines with regards to dosing schedule; criteria for dose reduction, interruption, or discontinuation.

### Endpoints and outcome assessments

The primary endpoint was overall survival (not reported in this preliminary analysis). Secondary endpoints assessed in this preliminary analysis included safety, and investigator (INV)-assessed overall response rate (ORR) and complete response rate (CRR) at EOT. Grade ≥ 3 AEs, and AEs leading to polatuzumab vedotin discontinuation, were collected. Severity of AEs was determined according to the National Cancer Institute Common Terminology Criteria for Adverse Events (CTCAE) version 5.0) [16]. The observation period for AEs was from the start of treatment until 90 days after the end of protocol treatment or until the start of subsequent therapy, whichever occurred first. INV-assessed ORR and CRR at EOT were based on the results of the first evaluation after completion of treatment, and were assessed based on the Lugano Classification of response assessment for lymphoma [17]. Modalities of assessment were determined by the physician; positron emission tomography or computed tomography were not mandatory. Safety and efficacy in the context of patient characteristics as well as relative dose intensity (the proportion of actual administered dose relative to planned dose; see Supplementary Methods for further details) and geriatric functional assessment (assessed using the Geriatric [G]8 survey at baseline in patients ≥ 65 years) were reported. The G8 survey was used to assess multiple domains including cognition, depression, disability, nutrition and comorbidities, with scores ranging from 0 (heavily impaired) to 17 (not at all impaired). A validated cut off value of 14 (> 14 versus ≤ 14) was set in this analysis [18].

### Statistical analysis

Continuous data are presented using summary statistics, including arithmetic and geometric means, median, range, standard deviations, and coefficients of variation. Categorical data are presented using summary statistics, including frequency and contingency tables. For patients whose baseline lesion information was available and who had responses assessed (efficacy-evaluable population), frequency and proportion of overall response at EOT were summarized, and EOT-CRR, EOT-ORR, and 95% confidence intervals (CIs) were calculated.

A target sample size of 500 patients was set without a predetermined hypothesis and based on feasibility. This enabled precise estimation of the 2-year survival rate in a patient population reflecting the real-world clinical environment in Japan. Among the 500 patients, 125 were estimated to relapse and receive second-line therapy within 2 years. Based on the POLARIX study and no censoring at time points up to 2 years, a 2-year survival rate of approximately 85%, with an estimated 95% CI of 81.8% to 88.1%, was assumed in this population. It was also estimated that 60 of the 500 enrolled patients would be aged > 80 years. Based on a previous study [19], assuming a 2-year survival rate of 60% in this population, the 95% CI was estimated as 47.6% to 72.4%.

## Results

### Patients

Between February 9, 2023 and December 6, 2023, a total of 502 patients were prospectively enrolled across 71 sites. At data cut-off (preliminary analysis: December 20, 2023), an initial 199 patients were eligible for this analysis (intention-to-treat), and 192 patients were included in the full analysis set (FAS). Reasons for exclusion from the FAS included: starting steroids, that were a component of Pola-R-CHP treatment prior to enrollment (*n* = 5), starting treatment before enrollment (*n* = 1), and study discontinuation (*n* = 1). Baseline demographic and clinical characteristics in the FAS and in patients aged > 80 years are shown in Table 1 and Supplementary Table 1, respectively. In the FAS, median age was 71.0 years (range 30–91) and 25 (13.0%) patients were aged > 80 years. Of the patients for whom ECOG PS and IPI was reported (*n* = 188), 179 (95.2%) had an ECOG PS of 0–2, and 134 (71.3%) had an IPI score of 2–5.

**Table 1:** Baseline demographic and clinical characteristics in the FAS

	FAS (*N* = 192)
Median age, years (range)	71.0 (30–91)
Age, n (%)	
< 65 years	57 (29.7)
≥ 65 to < 75 years	65 (33.9)
≥ 75 to < 85 years	59 (30.7)
≥ 85 years	11 (5.7)
Age > 80 years, n (%)	25 (13.0)
Sex, n (%)	
Male	109 (56.8)
Female	83 (43.2)
Clinical diagnosis, n (%)	
DLBCL	183 (95.3)
EBV-positive DLBCL, not otherwise specified	5 (2.6)
Intravascular large B-cell lymphoma	2 (1.0)
Grade 3b follicular lymphoma	2 (1.0)
Transformed low-grade lymphoma, n (%)	
Yes	9 (4.7)
No	183 (95.3)
ECOG PS, n (%)^a^	*n* = 188
0	119 (63.3)
1	47 (25.0)
2	13 (6.9)
3	6 (3.2)
4	3 (1.6)
Ann Arbor Stage, n (%)	
I	26 (13.6)
II	50 (26.0)
III	36 (18.7)
IV	80 (41.7)
IPI, n (%)^a^	*n* = 188
Low (0, 1)	54 (28.7)
Low–intermediate (2)	50 (26.6)
High–intermediate (3)	50 (26.6)
High (4, 5)	34 (18.1)
G8, n (%)^b^	*n* = 133
≤ 14	95 (71.4)
> 14	38 (28.6)
Bulky disease (≥ 7.5cm), n (%)	
Yes	28 (14.6)
No	164 (85.4)
LDH level, n (%)	
≤ ULN	91 (47.4)
> ULN	101 (52.6)
Bone marrow involvement, n (%)^c^	*n* = 186
Yes	14 (7.5)
No	172 (92.5)
Involved extranodal sites, n (%)	
0–1	137 (71.4)
≥ 2	55 (28.6)
Median time from initial diagnosis to treatment initiation, days (range)	26.5 (4.0–327.0)
Double-expressor lymphoma, n (%)^d^	*n* = 85
	36 (42.4)
Double-hit or triple-hit lymphoma, n (%)^e^	*n* = 21
	4 (19.0)

*DLBCL* diffuse large B-cell lymphoma, *EBV* Epstein‒Barr virus, *ECOG PS* Eastern Cooperative Oncology Group performance status, *FAS* full analysis set, *G8* Geriatric 8 survey, *IHC* immunohistochemistry, *IPI* International Prognostic Index, *LDH* lactate dehydrogenase, *ULN* upper limit of normal

^a^ECOG PS was not reported in four patients. Due to missing data, IPI was also not reported for these patients

^b^No entry in electronic data capture

^c^No entry in electronic data capture

^d^Patients with co-expression of MYC and BCL2, as determined by IHC analysis, were regarded as double expressors. Patients with unknown IHC results were excluded

^e^Double-hit included patients positive for MYC and BCL2 or MYC and BCL6. Triple-hit included patients positive for MYC, BCL2 and BCL6. Patients with unknown MYC, BCL2 or BCL6 were excluded

Overall, 163 (88.1%) patients completed planned treatment with Pola-R-CHP, and the median time from initial diagnosis to treatment initiation was 26.5 days (range 4.0–327.0). In total, 22 (11.9%) patients prematurely discontinued Pola-R-CHP treatment, three of whom were aged > 80 years. The reasons for treatment discontinuation were AEs (*n* = 10), disease progression (*n* = 3), death (*n* = 2) and other (*n* = 7; changing hospital [*n* = 1], patient request [*n* = 2], refusal to continue treatment [*n* = 1], treatment of lung cancer [*n* = 1], assessed as CR after the onset of pneumonia [*n* = 1], activities of daily living decreased [*n* = 1]). At data cut-off, treatment was ongoing for seven patients.

The median initial dose of polatuzumab vedotin was 1.8 mg/kg (range 1.0–1.9) for both patients in the FAS and for those > 80 years (Supplementary Table 2). The median relative dose intensity in the FAS was 94.8% for rituximab, 93.2% for polatuzumab vedotin, 88.0% for cyclophosphamide, 87.5% for doxorubicin, and 90.3% for prednisolone (Table 2). Most patients aged > 80 years received reduced doses from the first cycles; in this patient subgroup, the median relative dose intensity was 96.4% for rituximab, 95.0% for polatuzumab vedotin, 50.3% for cyclophosphamide, 49.2% for doxorubicin, and 56.4% for prednisolone (Supplementary Table 3).

**Table 2:** Relative dose intensity for treatments received in the FAS

	Drug	Mean ± SD	Median (range)
RDI (%)	Rituximab	92.8 ± 11.65	94.8 (44.3–125.4)
	Polatuzumab vedotin	89.4 ± 13.54	93.2 (15.6–114.0)
	Cyclophosphamide	80.9 ± 18.84	88.0 (7.4–103.7)
	Doxorubicin	79.9 ± 20.98	87.5 (0.0–103.2)
	Prednisolone	79.4 ± 22.63	90.3 (10.0–100.8)
Cycles	Rituximab	6.4 ± 1.50	6.0 (1.0–8.0)
	Polatuzumab vedotin	5.6 ± 1.05	6.0 (1.0–6.0)
	Cyclophosphamide	5.6 ± 1.04	6.0 (1.0–6.0)
	Doxorubicin	5.6 ± 1.16	6.0 (0.0–6.0)
	Prednisolone	5.6 ± 1.05	6.0 (1.0–6.0)

*FAS* full analysis set, *RDI* relative dose intensity, *SD* standard deviation

### Safety

Grade ≥ 3 AEs occurred in 99 (51.6%) patients in the FAS and 19 (76.0%) patients aged > 80 years (Table 3). Hematologic events were the most frequently occurring AEs in the FAS, including neutrophil count decreased (*n* = 54; 28.1%), white blood cell decreased (*n* = 22; 11.5%), and febrile neutropenia (*n* = 19; 9.9%). Febrile neutropenia was numerically higher in patients > 80 years compared with the overall FAS. Serious AEs occurred in 29 (15.1%) patients in the FAS. When stratified by age (< 65 years vs ≥ 65 years), the incidence of any grade ≥ 3 AE in the FAS was approximately 10% higher in those aged ≥ 65 years (Table 4). In patients aged ≥ 65 years who completed the G8 survey (*n* = 133), those with a G8 score of ≤ 14 had a 10.5% higher occurrence of grade ≥ 3 AEs than those with a G8 score of > 14. AEs in patients aged < 70 years and ≥ 70 years are presented in Supplementary Table 4. The majority of AEs occurred in Cycle 1 and were hematologic; median time to first occurrence was 12.5 days (range 9.0–167.0) for neutrophil count decreased, 12.5 days (range 8.0–104.0) for white blood cell decreased, and 12.0 days (range 7.0–147.0) for febrile neutropenia.

**Table 3:** Grade ≥ 3 AEs in the FAS and patients aged > 80 years

n (%)	FAS (*N* = 192)	> 80 years (*n* = 25)
All events grade ≥ 3	99 (51.6)	19 (76.0)
Neutrophil count decreased	54 (28.1)	7 (28.0)
White blood cell decreased	22 (11.5)	3 (12.0)
Febrile neutropenia	19 (9.9)	6 (24.0)
Anemia	12 (6.3)	3 (12.0)
Platelet count decreased	9 (4.7)	2 (8.0)
Lung infection	8 (4.2)	3 (12.0)
Infections and infestations, others (COVID-19 infection)	5 (2.6)	2 (8.0)
Sepsis	2 (1.0)	2 (8.0)

*AEs* adverse events, *COVID-19* coronavirus disease 2019, *FAS* full analysis set

**Table 4:** Grade ≥ 3 AEs in patients aged < 65 vs ≥ 65 years, and those aged ≥ 65 years with G8 survey results (G8 score > 14 vs ≤ 14)

n (%)	< 65 years (*n* = 57)	≥ 65 years (*n* = 135)	≥ 65 years (*n* = 133)	
			G8 > 14 (*n* = 38)	G8 ≤ 14 (*n* = 95)
All events	26 (45.6)	73 (54.1)	18 (47.4)	55 (57.9)
Neutrophil count decreased	17 (29.8)	37 (27.4)	10 (26.3)	27 (28.4)
White blood cell decreased	9 (15.8)	13 (9.6)	4 (10.5)	9 (9.5)
Febrile neutropenia	3 (5.3)	16 (11.9)	3 (7.9)	13 (13.7)
Anemia	0 (0.0)	12 (8.9)	3 (7.9)	9 (9.5)
Platelet count decreased	1 (1.8)	8 (5.9)	3 (7.9)	5 (5.3)
Lung infection	1 (1.8)	7 (5.2)	1 (2.6)	6 (6.3)
Lymphocyte count decreased	2 (3.5)	5 (3.7)	3 (7.9)	2 (2.1)

*AE* adverse event, *G8* Geriatric 8

Fifteen (7.8%) patients in the FAS discontinued polatuzumab vedotin treatment due to AEs (Table 5) and, of these, four patients were aged > 80 years. The most common AEs leading to polatuzumab vedotin discontinuation included peripheral sensory neuropathy (*n* = 3), lung infection (*n* = 2), and COVID-19 infection (*n* = 2). Two deaths were reported, one of which was due to sepsis (patient was aged > 80 years) and the other due to pneumonitis (patient was aged 75–80 years).

**Table 5:** AEs leading to polatuzumab vedotin discontinuation in the FAS

n (%)	FAS (*N* = 192)
All events	15 (7.8)
Peripheral sensory neuropathy	3 (1.6)
Lung infection	2 (1.0)
Infections and infestations, others (COVID-19 infection)	2 (1.0)
Constipation	1 (0.5)
Malaise	1 (0.5)
Sepsis	1 (0.5)
Hip fracture	1 (0.5)
Fibrinogen decreased	1 (0.5)
Neutrophil count decrease	1 (0.5)
Weight gain	1 (0.5)
Anorexia	1 (0.5)
Pneumonitis	1 (0.5)

*AE* adverse event, *COVID-19* coronavirus disease 2019, *FAS* full analysis set

### Response to treatment

In the efficacy-evaluable population at EOT (*n* = 141; Table 6), ORR was 95.0% (95% CI, 90.1–97.6), and CRR was 87.9% (95% CI, 81.5–92.3). Overall, 121 (85.8%) evaluable patients achieved complete response, 10 (7.1%) achieved partial response, and one (0.7%) achieved stable disease. Five (3.5%) patients had progressive/relapsing disease. In efficacy-evaluable patients aged > 80 years (*n* = 21), the EOT-ORR was 90.5% (95% CI, 71.1–97.3) and the EOT-CRR was 85.7% (95% CI, 65.4–95.0).

**Table 6:** Investigator-assessed EOT response assessments

n (%)	Efficacy-evaluable patients (*N* = 141)
CRR [95% CI]	124 (87.9) [81.5–92.3]
ORR [95% CI]	134 (95.0) [90.1–97.6]
CR	121 (85.8)
Cru	3 (2.1)
PR	10 (7.1)
SD	1 (0.7)
PD/RD	5 (3.5)
NE	1 (0.7)
Missing	0 (0.0)

*CI* confidence interval, *CR* complete response, *CRu* complete response unconfirmed, *CRR* complete response rate (CR + CRu), *EOT* end-of-treatment, *NE* not evaluable, *ORR* overall response rate (CR + CRu + PR), *PD* progressive disease, *PR* partial response, *RD* relapsing disease, *SD* stable disease

## Discussion

In this preliminary analysis of the POLASTAR study, we have shown that the initial safety and efficacy data of Pola-R-CHP in patients with previously untreated DLBCL were consistent with the previously reported primary findings of the global POLARIX study [12]. Safety data were similar between studies, with no new safety signals identified in this analysis.

This real-world study included patients who were excluded from POLARIX [12]. Overall, patients in this study were older compared with patients treated with Pola-R-CHP in POLARIX (median age 71 years vs 65 years [12]), as our analysis included patients aged > 80 years versus 18–80 years in POLARIX. In addition, this study included patients with low IPI scores (0–1; 28.7%), whereas POLARIX enrolled patients with higher IPI scores (2–5), showing that Pola-R-CHP is a selected treatment option for patients at low risk in real-world clinical practice despite the Japanese guidelines recommendation of 4 cycles of R-CHOP followed by 2 cycles of rituximab monotherapy for patients younger than 60 years, with limited stage DLBCL without a bulky mass (> 7.5 cm), and with an age-adjusted IPI of 0 [13, 14]. The clinical significance of Pola-R-CHP in patients with an IPI score of 0–1 cannot be determined with the current short follow-up time and further investigation in the future is needed. Furthermore, our study also included patients with ECOG PS ≥ 3, unlike the POLARIX study, which limited inclusion to those with ECOG PS 0–2 only; the proportion of patients with ECOG PS ≥ 3 was very small (4.8%) in this study. In our study, the majority of patients were able to complete the protocol treatment, which is consistent with the primary analysis of POLARIX [12, 15]. Overall, broadening our inclusion criteria enabled better reflection of the real-world population with DLBCL in Japan.

The safety profile of the Pola-R-CHP regimen was comparable across the POLASTAR and POLARIX studies, with both reporting hematologic events as the most frequently occurring AEs [12]. In our study, the most common grade ≥ 3 AEs for patients in the FAS were neutrophil count decreased, white blood cell decreased, febrile neutropenia, and anemia. Similarly, the most common grade 3/4 AEs observed with Pola-R-CHP in POLARIX were neutropenia, febrile neutropenia, and anemia [12]. In patients aged > 80 years, the most frequent grade ≥ 3 AEs were comparable with those presented by patients in the FAS. Nonetheless, patients aged > 80 years presented with more toxicities, with the rates of febrile neutropenia (24.0% vs 9.9%) and anemia (12.0% vs 6.3%) being numerically higher compared with patients in the FAS despite more frequent initial dose reductions (Supplementary Table 2). The European phase III POLAR BEAR study, which was designed to compare two attenuated regimens (R-mini-CHOP versus Pola-R-mini-CHP) for patients with previously untreated DLBCL aged > 80 years, or 75–80 years and frail (*N* = 127), showed that both treatment options were tolerable in an elderly population [20]. Overall, polatuzumab vedotin-containing reduced dose regimens may be viable treatment options for patients with previously untreated DLBCL aged > 80 years.

With regards to efficacy, we evaluated EOT-ORR and EOT-CRR in this analysis. Other efficacy endpoints including progression-free survival, best overall response, and overall survival were not assessed in this analysis due to the short follow-up period. INV-assessed EOT-ORR (95.0%) and EOT-CRR (87.9%) were similar in patients in the FAS and those receiving Pola-R-CHP in POLARIX (independent central review-assessed EOT-ORR, 85.5%; EOT-CRR, 78.0%) [12]. Our initial efficacy results were also similar to those observed in a single-institute, retrospective, observational study of Pola-R-CHP in Japan (*N* = 30; aged > 80 years, *n* = 8), which demonstrated an ORR of 93.3% and a CRR of 86.7% at EOT [21]. Our findings demonstrate that high responses can be achieved with Pola-R-CHP in a large Japanese patient population with previously untreated DLBCL. To further assess efficacy and risk of early relapse, exploratory evaluation of cell-of-origin, genetic subtypes, CD79b expression, and ctDNA levels in blood samples is planned.

A key strength of this study is the inclusion of patients who were not eligible for the POLARIX study, reflecting a diverse patient cohort that is more representative of real-world clinical practice compared with clinical trials. Furthermore, real-world data from patients aged > 80 years enrolled in this analysis support Pola-R-CHP as a first-line treatment option for elderly patients with DLBCL.

One limitation of the POLASTAR study is that only grade ≥ 3 AEs and any-grade AEs leading to polatuzumab vedotin discontinuation were collected. In addition, as this prospective observational study was designed to collect real-world, clinical information, there was no central evaluation for efficacy; the timing and modality of assessment was determined by the investigator. Exploratory analysis in elderly patients with DLBCL was limited by the small sample size for the elderly population; however, the data were comparable with the younger population (≤ 80 years) in this analysis.

## Conclusion

Even though the POLASTAR study enrolled a diverse patient cohort including those who were ineligible for the POLARIX study, initial results from this study were consistent with previously published data from POLARIX. The findings suggest that Pola-R-CHP may be effective and safe for patients with previously untreated DLBCL in the real-world population. The recruitment of 500 patients has been completed and analyses are ongoing.

## Supplementary Information

Below is the link to the electronic supplementary material.

Supplementary file1 (DOCX 57 KB)

## Data Availability

A summary of the study is registered in the Japan Registry of Clinical Trials (jRCT). Planned recruitment of 500 patients was completed in November 2023, and study results will be posted at the following link promptly after completion of the study: https://jrct.niph.go.jp/en-latest-detail/jRCT1071220082.

## Declarations

### Permission to reproduce material from other sources

N/A
