# Supplementary material for: Preliminary analysis of a multicenter study of Pola-R-CHP in untreated Japanese patients with DLBCL (POLASTAR)
Source: Int J Hematol. 2025 Dec 3;123(3):404–11. doi: 10.1007/s12185-025-04122-w (PMC12967436; doi:10.1007/s12185-025-04122-w)
Supplement: Supplementary file 1 — Supplementary file1 (DOCX 57 KB) [file 12185_2025_4122_MOESM1_ESM.docx]

**Supplementary material**

**Preliminary analysis of a multicenter study of Pola-R-CHP in untreated Japanese patients with DLBCL (POLASTAR)**

**Supplementary Methods**

**Calculation of relative dose intensity (RDI)**

RDI = $\frac{Actual dose}{Expected dose}\times100$

*Where:*

*Actual dose* = $\frac{Actual total dose}{Actual total duration of treatment}$

*Expected dose* = $\frac{Total planned dose}{Total planned duration of treatment}$

*Total planned dose* = $\frac{Maximum dose allowed in the protocol \times maximum number of cycles allowed in the protocol}{Total planned duration of treatment}$

**Supplementary Table 1** Baseline demographic and clinical characteristics in patients aged >80 years

|  | **Aged >80 years**  **(*n* = 25)** |
| --- | --- |
| **Median age (range)** | 84.0 (81–91) |
| **Sex, n (%)**  Male  Female | 17 (68.0)  8 (32.0) |
| **Clinical diagnosis, n (%)**  DLBCL  EBV-positive DLBCL, not otherwise specified  Intravascular large B-cell lymphoma  Grade 3b follicular lymphoma | 23 (92.0)  2 (8.0)  0 (0.0)  0 (0.0) |
| **Transformed low-grade lymphoma, n (%)**  Yes  No | 1 (4.0)  24 (96.0) |
| **ECOG PS, n (%)**  0  1  2  3  4 | 15 (60.0)  7 (28.0)  1 (4.0)  1 (4.0)  1 (4.0) |
| **Ann Arbor stage, n (%)**  I  II  III  IV | 5 (20.0)  8 (32.0)  4 (16.0)  8 (32.0) |
| **IPI, n (%)**  Low (0,1)  Low–intermediate (2)  High–intermediate (3)  High (4,5) | 3 (12.0)  10 (40.0)  8 (32.0)  4 (16.0) |
| **G8, n (%)**  ≤14  >14 | 21 (84.0)  4 (16.0) |
| **Bulky disease (≥7.5cm), n (%)**  Yes  No | 1 (4.0)  24 (96.0) |
| **LDH level, n (%)**  ≤ULN  >ULN | 8 (32.0)  17 (68.0) |
| **Bone marrow involvement, n (%)**  Yes  No | 2 (8.0)  23 (92.0) |
| **Involved extranodal sites, n (%)**  0–1  ≥2 | 19 (76.0)  6 (24.0) |
| **Median time from initial diagnosis to treatment initiation, days (range)** | 26.0 (13.0–76.0) |
| **Double-expressor lymphoma, n (%)^a^** | *n* = 10  5 (50.0) |
| **Double-hit or triple-hit lymphoma, n (%)^b^** | *n* = 4 1 (25.0) |

*DLBCL* diffuse large B-cell lymphoma, *EBV* Epstein-Barr virus, *ECOG PS* Eastern Cooperative Oncology Group performance status, *G8* Geriatric 8 survey. *IPI* International Prognostic Index, *LDH* lactate dehydrogenase, *ULN* upper limit of normal

^a^Patients with co-expression of MYC and BCL2, as determined by IHC analysis, were regarded as double expressors. Patients with unknown IHC results were excluded

^b^Double-hit included patients positive for MYC and BCL2 or MYC and BCL6. Triple-hit included patients positive for MYC, BCL2 and BCL6. Patients with unknown MYC, BCL2 or BCL6 were excluded

**Supplementary Table 2** Initial doses administered in FAS, patients aged ≤80 years and those aged >80 years

| **Drug** | **FAS** **(*N* = 192)** | | | **≤80 years (*n* = 167)** | | **>80 years (*n* = 25)** | | |
| --- | --- | --- | --- | --- | --- | --- | --- | --- |
|  | **Mean ± SD** | **Median (range)** | **Mean ± SD** | | **Median (range)** | | **Mean ± SD** | **Median (range)** |
| **Rituximab (mg/m^2^)** | 373.6 ± 13.40 | 373.8  (298.2–418.5) | 373.7 ± 13.5 | | 373.8  (298.2–418.5) | | 373.4 ± 12.7 | 373.8  (335.7–406.7) |
| **Polatuzumab vedotin (mg/kg)** | 1.8 ± 0.12 | 1.8 (1.0–1.9) | 1.8 ± 0.1 | | 1.8 (1.1–1.9) | | 1.7 ± 0.2 | 1.8  (1.0–1.9) |
| **Cyclophosphamide (mg/m^2^)** | 668.5 ± 123.17 | 732.3  (304.7–764.5) | 702.2 ± 83.8 | | 739.3  (332.4–764.5) | | 443.8 ± 107.8 | 398.6  (304.7–730.1) |
| **Doxorubicin (mg/m^2^)** | 44.0 ± 9.45 | 48.5  (0.0–51.6) | 46.2 ± 7.6 | | 49.0 (0.0–51.6) | | 29.2 ± 7.2 | 25.0  (22.8–48.7) |
| **Prednisolone (mg)^a^** | 88.7 ± 19.58 | 100.0  (20.0–100.0) | 92.1 ± 16.7 | | 100.0  (30.0–100.0) | | 65.8 ± 22.1 | 60.0  (20.0–100.0) |

*FAS* full analysis set, *SD* standard deviation

^a^*N* = 191; excluding one patient (≤80 years old) administered with methylprednisolone 80.0 mg

**Supplementary Table 3** Relative dose intensity for treatments received by patients aged >80 years

|  | **Drug** | **>80 years**  **(*n* = 25)** | |
| --- | --- | --- | --- |
|  |  | **Mean ± SD** | **Median (range)** |
| **RDI (%)** | **Rituximab** | 92.6 ± 13.3 | 96.4 (44.3–109.7) |
|  | **Polatuzumab vedotin** | 89.0 ± 15.2 | 95.0 (42.9–105.7) |
|  | **Cyclophosphamide** | 52.0 ± 16.5 | 50.3 (7.4–85.5) |
|  | **Doxorubicin** | 51.2 ± 16.6 | 49.2 (7.8–84.9) |
|  | **Prednisolone** | 56.9 ± 22.1 | 56.4 (10.0–100.0) |
| **Cycles** | **Rituximab** | 6.4 ± 1.4 | 6.0 (3.0–8.0) |
|  | **Polatuzumab vedotin** | 5.7 ± 0.9 | 6.0 (3.0–6.0) |
|  | **Cyclophosphamide** | 5.5 ± 1.3 | 6.0 (1.0–6.0) |
|  | **Doxorubicin** | 5.5 ± 1.3 | 6.0 (1.0–6.0) |
|  | **Prednisolone** | 5.5 ± 1.3 | 6.0 (1.0–6.0) |

*RDI* relative dose intensity, *SD* standard deviation

**Supplementary Table 4** Grade ≥3 AEs in patients aged <70 years and ≥70 years

| **n (%)** | **<70 years**  **(*n* = 86)** | **≥70 years**  **(*n* = 106)** |
| --- | --- | --- |
| **All events** | 39 (45.3) | 60 (56.6) |
| **Neutrophil count decreased** | 23 (26.7) | 31 (29.2) |
| **White blood cell decreased** | 12 (14.0) | 10 (9.4) |
| **Febrile neutropenia** | 4 (4.7) | 15(14.2) |
| **Anemia** | 2 (2.3) | 10(9.4) |
| **Platelet count decreased** | 2 (2.3) | 7(6.6) |
| **Lung infection** | 1 (1.2) | 7(6.6) |

*AE* adverse event
